# Stimulation of Cardiomyocyte Proliferation Is Dependent on Species and Level of Maturation

**DOI:** 10.3389/fcell.2022.806564

**Published:** 2022-05-19

**Authors:** Dogacan Yücel, Bayardo I. Garay, Rita C. R. Perlingeiro, Jop H. van Berlo

**Affiliations:** ^1^ Department of Medicine, Lillehei Heart Institute, Cardiovascular Division, University of Minnesota, Minneapolis, MN, United States; ^2^ Department of Integrative Biology and Physiology, University of Minnesota, Minneapolis, MN, United States; ^3^ Department of Neuroscience, University of Minnesota, Minneapolis, MN, United States; ^4^ Medical Scientist Training Program, University of Minnesota Medical School, Minneapolis, MN, United States; ^5^ Stem Cell Institute, University of Minnesota, Minneapolis, MN, United States

**Keywords:** cardiomyocyte, proliferation, cell cycle, live-cell imaging, CDKN1A (p21)

## Abstract

The heart is one of the least regenerative organs. This is in large part due to the inability of adult mammalian cardiomyocytes to proliferate and divide. In recent years, a number of small molecules and molecular targets have been identified to stimulate cardiomyocyte proliferation, including p38 inhibition, YAP-Tead activation, fibroblast growth factor 1 and Neuregulin 1. Despite these exciting initial findings, a therapeutic approach to enhance cardiomyocyte proliferation *in vivo* is still lacking. We hypothesized that a more comprehensive *in vitro* validation using live-cell imaging and assessment of the proliferative effects on various cardiomyocyte sources might identify the most potent proliferative stimuli. Here, we used previously published stimuli to determine their proliferative effect on cardiomyocytes from different species and isolated from different developmental timepoints. Although all stimuli enhanced DNA synthesis and Histone H3 phosphorylation in neonatal rat ventricular cardiomyocytes to similar degrees, these effects varied substantially in mouse cardiomyocytes and human iPSC-derived cardiomyocytes. Our results highlight p21 inhibition and Yap-Tead activation as potent proliferative strategies to induce cultured cardiomyocyte cell cycle activity across mouse, rat and human cardiomyocytes.

## Introduction

Cardiovascular disease (CVD) is the leading cause of death in Western society (Virani et al., 2020). Although treatments for acute ischemic events have improved outcomes for patients over the past 40 years, the prevalence for heart failure is continuing to increase. This is in part due to the inability of adult cardiomyocytes to regenerate lost myocardium, and the lack of progenitor cells in the adult heart that could repopulate lost cardiomyocytes. At least two independent strategies to achieve cardiac regeneration, and increase the cardiomyocyte content of the heart, are currently explored in basic and translational science experiments. The first approach aims to exogenously add cells to the heart that are pre-differentiated into cardiac progenitor cells or cardiomyocytes. This approach is currently in pre-clinical testing, and involves expansion, differentiation and integration of cardiac progenitor cells or their derivatives into the heart (Shiba et al., 2012; Chong et al., 2014; Shiba et al., 2016). Although initial studies demonstrated promising results, human induced pluripotent stem cell derived cardiomyocytes (hiPSC-CMs) are not yet approved for use in patients in the US, and were observed to cause ventricular tachycardias in large animal experiments (Povsic et al., 2011; Shiba et al., 2016). However, it is important to note that although hiPSC-CM based treatments have potential issues, they are important for disease modeling, and moreover, transplantation in animal models resulted in enhanced cardiac function (Jabbour et al., 2021; Kawaguchi et al., 2021). Regardless, a number of clinical trials are currently underway to assess the safety and efficacy of transplantation of cardiomyocytes in various forms (NTC03763136, NTC04396899, NTC04696328, NTC04945018, and NTC04982081).

The other approach that is being investigated, but not yet in pre-clinical testing, is based on direct stimulation of cardiomyocyte proliferation (Tzahor and Poss, 2017). It has previously been established by various groups that cardiomyocytes during embryonic development are proliferative and capable of completing cell cycle. However, soon after birth, cardiomyocytes undergo a final round of DNA synthesis, resulting in endoreplication, giving rise to polyploid, and terminally differentiated cardiomyocytes (Soonpaa et al., 1996; Soonpaa and Field, 1997). Most mammals show these terminal endoreplication events in as many as 95% of their cardiomyocytes, although the level of mononucleated diploid ones could show variation, and this variation might underlie differences in cardiac regeneration (Steinhauser and Lee, 2011). In part due to their terminal polyploid differentiated state, cardiomyocytes are one of the least regenerative cell types in the mammalian body. This is in stark contrast to zebrafish and newt, which show reactivation of cell cycle in adult cardiomyocytes, to give rise to completely regenerated hearts in response to cardiac injury (Oberpriller and Oberpriller, 1974; Poss et al., 2002). Importantly, neonatal mice also have the ability to completely recover from cardiac injury, but only when that injury is induced at a time when cardiomyocytes are still proliferative and diploid, i.e., in the first few days of life. In addition to neonatal mouse, other studies in zebrafish demonstrated that complete regeneration in these animals is achieved by proliferation of resident cardiomyocytes instead of progenitor cells (Kikuchi et al., 2010; Gupta and Poss, 2012). These studies show that enhancing cardiomyocyte proliferation might lead to a therapeutic strategy to repair the heart after injury. In the past decade, a number of regulators of cardiomyocyte proliferation have been identified, with most recently a combination of 4 different cell cycle regulators to enhance cardiomyocyte proliferation both *in vitro* and *in vivo* (Magadum et al., 2017; Nakada et al., 2017; Zhang and Del Re, 2017; Mohamed et al., 2018).

Although a number of compounds and genes have been identified to enhance cardiomyocyte proliferation, none of these have been translated to therapies thus far. There are many potential reasons for why that might be. Probably one of the most important factors is that the *in vitro* environment, where compounds are initially identified, is very different from the *in vivo* environment. However, even experiments performed *in vitro* face challenges. First, primary cultures that are inherently not pure for cardiomyocytes have to be used for *in vitro* analyses. The final assessment performed a number of days after isolation could be a mixture of enhanced survival, and proliferation. Another major hurdle is the difference in cardiomyocyte proliferation in neonatal cells vs. adult cells. Showing that an agent actually stimulates cardiomyocyte proliferation needs very rigorous validation because cytokinesis and formation of new cardiomyocytes are not easily assessed with a single assay. To aid in the identification of treatments that can enhance cardiomyocyte proliferation, we determined the effect of published proliferative factors on cardiomyocytes derived from various species at different developmental timepoints to identify conditions that might be most conducive to proliferation stimulation.

## Materials and Methods

### Cardiomyocyte Culture

All animal procedures were performed in accordance with institutional guidelines and were approved by the University of Minnesota Institutional Animal Care and Use Committee. The abdomen and thorax of 1-day old rat pups (Sprague-Dawley, Charles River) were sterilized with 70% ethanol solution and pups were euthanized by decapitation. The hearts of neonatal pups were surgically removed, and cardiomyocytes were isolated using the Worthington Neonatal Cardiomyocyte Isolation System according to the manufacturer’s protocol. For mouse embryonic timepoints, we shortened the trypsin incubation from overnight to 2 h and followed rest of the protocol as instructed in the manual. For neonatal mouse culture, we digested hearts in Trypsin containing HBSS (1.75 mg/ml) (Difco, 215250) under continuous stirring for 1.5 h while collecting the supernatant containing cells every 8 min. Following isolation, cells were pre-plated on un-coated dishes for 1 h to remove fibroblasts, and non-adherent cells were then seeded onto 0.1% gelatin (Bio-Rad, 170-6537) coated dishes. We used 96-well plates (Corning 3340) for staining, and live-cell imaging assays and 6-well plates for qPCR and western blotting. Cardiomyocytes were plated in DMEM (Thermo Fisher, 10013CV) with 10% fetal bovine serum (Fisher Scientific, SH3007103), 5% horse serum (Gibco, 26050088) and 1% penicillin streptomycin (P/S) (Fisher Scientific, MT300002CI). After 24 h, the media was changed to DMEM with 5% horse serum and 1% P/S. Thirty-6 hours after plating, adenoviruses were added at a multiplicity of infection (MOI) of 25–50 to specific conditions as indicated below. Twelve hours after the addition of viruses, the media was changed into serum free DMEM containing 1% P/S with the addition of growth factors (NRG1, 100 ng/ml, FGF1 50 ng/ml (R&D Systems diluted in 0.1% BSA/PBS)) or small molecules (SB203580, 10 µM (Cell Signaling), TT-10, 5 µM (Sigma)) as indicated. For adult mouse cardiomyocytes, we used media containing chemically defined lipid concentrate as described previously (Ackers-Johnson and Foo, 2019). For hiPSC-CMs, we administered factors in RPMI with B27 supplement minus insulin. Media for all samples was replenished every 24 h thereafter with the appropriate growth factors or small molecules. For some experiments we cultured cardiomyocytes in the presence of serum, as indicated within the results section. For transfection, lipofectamine RNAiMAX (Thermo Fisher Scientific, 13778075) was used according to manufacturer’s instructions to deliver p21 (human siRNA, Santa Cruz Biotechnology; sc-29427 and mouse siRNA, Dharmacon, L-058636-00-0005) or scrambled siRNAs (Dharmacon, D-001810-10-20). To measure DNA synthesis, 5-ethynyl-2′-deoxyuridine (EdU, 10 µM (Carbosynth)) was added 24–48 h before fixation.

### Adenovirus

Adenoviruses to specifically express a cardiomyocyte nuclear marker were generated to contain the rat troponin T promoter to obtain cardiomyocyte specific expression (Wang et al., 1994). This promoter is followed by an mCherry tagged Histone H2B complementary DNA to allow live-cell visualization of cardiomyocyte nuclei. This adenoviral construct was purchased from Vectorbuilder. Adenoviruses containing anti Cdkn1a short-hairpin RNA (shRNA) or scrambled shRNA were generated by annealing synthetic shRNAs (IDT) and ligating them into the U6 promoter containing BLOCK-iT adenoviral vector (Life Technologies). Viruses were amplified by transducing HEK293T cells (ATCC) and titered using an Adeno-X rapid titer test (Clontech) following the manufacturer’s protocol. Adenoviruses were used at an MOI of 25–50.

### Immunocytochemistry

Cardiomyocytes were fixed for immunocytochemistry using 4% PFA (Electron Microscopy Sciences, 15714) in PBS for 15 min at room temperature. For EdU staining, permeabilization was done with 0.5% Triton-X100 (Sigma-Aldrich, T8787) in PBS for 20 min, followed by 3% BSA in PBS twice for 10 min, and EdU labeling using 20 mM Tris. HCl pH8.5, 10 mM CuSO4, 10 µM Sulforhodamine B azide (AZ106 Click Chemistry Tools), and Sodium Ascorbate as reaction conditions for 20 min at room temperature. After washing twice in PBS we proceeded with immunocytochemistry using primary antibodies Troponin-T Cardiac Isoform Ab-1 (Fisher Scientific, MS295PABX, 1:400 dilution), phospho-Histone H3 Ser10 (Santa Cruz Biotechnology, sc-8656-R, 1:400 dilution) or Aurora Kinase B (Abcam, ab2254, 1:400 dilution) and secondary antibodies Alexa Fluor 488 Donkey Anti-Mouse (Life Technologies, A21202, 1:500 dilution) and Alexa Fluor 568 Donkey Anti-Rabbit (Life Technologies, A10042, 1:500 dilution) have been used along with DAPI (Life Technologies, D3571, and 200 ng/ml final concentration). To stain for live/dead cells, we performed live/dead staining assays on day 5. Plates were spun down and cells were incubated with 4 µM Calcein (Sigma-Aldrich, 17783) and 4 µM ethidium homodimer (Sigma Aldrich, 46063) for 1 h, followed by fixation and antibody staining for Troponin T with secondary antibody Alexa Fluor 647 Donkey Anti-Mouse (Life Technologies, A31571, 1:500 dilution).

### Adult Cardiomyocyte Culture

Adult cardiomyocytes were isolated from 3-month old C57Bl/6j or p21KO animals (JAX stock #016565) as previously described (Deng et al., 1995; Ackers-Johnson and Foo, 2019). Cardiomyocytes were seeded on laminin (Thermo Fisher Scientific, 23017015) coated 96-wells plates and cultured for 24 h before introducing EdU. Cells were then cultured for an additional 24 h, fixed and stained for EdU, DAPI and troponin T.

### Human iPSC Maintenance

We used 3 previously published hiPSC cell lines in this study; PLZ (Darabi et al., 2012), MYL2*-*mEGFP (Roberts et al., 2019), and DF 19-9-11T (Wheelwright et al., 2018). Cells were maintained in mTeSR™-1 medium (STEMCELL Technologies) on growth factor-reduced (GFR) Matrigel (∼8 μg/cm^2^, Corning) at 5% CO_2_ and 37°C under normoxia conditions. Cells were singularized with Accutase (Innovative Cell Technologies, Inc.) for 3–5 min and passaged every 4 days at a density of 1.25 × 10^4^/cm^2^ with the addition of 10 μM ROCK inhibitor (Y-27632, Cayman Chemical) for the first 24 h after each passage. Cell lines were routinely tested for *mycoplasma* contamination every 3 months (Applied Biological Materials, Inc.) following the manufacturer’s recommendation.

### Cardiac Differentiation by the Wnt Modulation Method

After passaging freshly thawed hiPSCs three times in single cell suspension using standard culture methods, CMs were generated using the Wnt modulation protocol (Lian et al., 2013). Briefly, hiPSCs were cultured in 12-well plates to a confluency of 70–85% over 3 days in Growth Factor Reduced-Matrigel (∼22 μg/cm^2^), and were induced with 6.5–8.5 μM CHIR 99021 (GSK3β inhibitor, Cayman Chemical) in RPMI1640 (Gibco) supplemented with B27 (minus insulin, Gibco). Exactly 48 h later, the media was completely removed and replaced with 7.5 μM IWP-2 (PORCN inhibitor, Cayman Chemical) in RPMI1640 supplemented with B27 (minus insulin). Following another 48-h period, the media was removed and replaced with RPMI1640 supplemented with B27 (minus insulin) on day 4 of the protocol. Beginning on day 6, the media was changed to RPMI1640 supplemented with B27 (plus insulin, Gibco) and changed every 48 h. Beating was observed between day 8–12 of the differentiation protocol. hiPSC-CMs were purified using DL-lactate to induce a metabolic switch, as previously described (Tohyama et al., 2013; Burridge et al., 2014). Briefly, on day 15 of the differentiation protocol, hiPSC-CMs were incubated with 0.25% trypsin in EDTA for 20 min, singularized, and quenched with three times the volume of trypsin in filtered RPMI1640 with 20% FBS (Sigma-Aldrich). Cells were centrifuged at 300 *g* for 5 min and resuspended in RPMI1640 supplemented with B27 (plus insulin) and 10 μM ROCK inhibitor. hiPSC-CMs were then seeded onto two 6-well plates per every 12-well plate (∼2.6x dilution by surface area). Forty-8 hours later, the media was changed to lactate media: RPMI1640 without D-glucose (Life Technologies), 5 mM sodium DL-lactate (L4263, Sigma-Aldrich), 213 μg/mL L-ascorbic acid 2-phosphate (Sigma-Aldrich), and 500 μg/ml *O. sativa-*derived recombinant human albumin (Sigma-Aldrich). The media was changed every 48 h for a total of 2–6 days upon which the media was reverted to RPMI1640 with B27 (plus insulin) supplement. Human iPSC-CMs were kept in culture for 70 days for mature and 28 days for immature hiPSC-CM cultures prior to replating. For p21 knockdown in hiPSC-CMs, cells were transfected with p21 siRNA (Santa Cruz Biotechnology, sc-29427) using lipofectamine RNAiMAX (Thermo Fisher Scientific, 13778075) based on manufacturer’s instructions for 12 h. The cell media was then changed into RPMI1640 with B27 (plus insulin) and corresponding growth factors or small molecules were added to the media based on the treatment.

### Imaging and Analysis

Images for live cell imaging were obtained using an inverted Nikon epifluorescent microscope equipped with a TiE motorized stage, a Lumencor SpectraX light engine and an Ibidi environmental control chamber available at the University Imaging Centers at the University of Minnesota. Imaging was performed in automated fashion using Nikon Jobs. Each well was imaged at 21 positions using a 10x objective (NA 0.25, working distance 7.5 mm). We avoided imaging edge areas. Images were tiled into a single image using ImageJ. Obtained images were tiled into a single image using ImageJ. We then used the ImageJ plugin Trackmate to identify cardiomyocyte nuclei (Tinevez et al., 2017). We used the Laplacian of Gaussian based segmentation (LoG) detector and adjusted the estimated blob diameter (expected size of H2B-mCherry positive nuclei in pixels) manually. This was done once since the size of nuclei was not expected to change substantially during the culture period. The threshold was adjusted manually to optimize H2B-mCherry detection, and thus of cardiomyocyte nuclei. For fixed cells we used a BioTek Cytation 3 Multi-Mode Reader and imaged 56 sites per well using a 10x objective. We tiled images into a single image using ImageJ, and used Cell Profiler to identify cell nuclei followed by determination of cardiomyocyte identity and EdU status (Carpenter et al., 2006; Bass et al., 2012). For measuring DNA content from DAPI, we combined previously published algorithms using CellProfiler (Roukos et al., 2015; Ackers-Johnson and Foo, 2019). Background subtraction was done using CorrectIlluminationCalculate and CorrectIlluminationApply functions. Thresholding for identifying nuclei objects were using Otsu Thresholding algorithm. We then measured masked Troponin and EdU images to measure mean intensity of TnnT and EdU within these nuclei to categorize nuclei as cardiomyocyte or non-cardiomyocyte and proliferating or non-proliferating ones. For ploidy analysis, we measured DAPI intensity from background subtracted DAPI images in individual nuclei objects (Yücel et al., 2020).

### qPCR

Total RNA was isolated from cardiomyocytes cultured in 6-well plates using Trizol (Life Technologies, 15596018). Isolated total RNA was quantified, and normalized amounts were used as template to produce cDNA using the SuperScript VILO cDNA Synthesis Kit (Thermo Fisher, 11754050). Relative abundance of cDNA was assessed by qPCR on an Applied Biosystems QuantStudio 6 Flex Real-Time PCR System using iTaq Universal SYBR Green Supermix (Bio-Rad, 1725124) and analyzed by using the delta-delta CT method. The following primers were used: Cdkn1a fw: 5′-GGG​ATG​CAT​CTA​TCT​TGT​GAT​ATG-3′, Cdkn1a rev: 5′-GTG​GAA​CAG​GTC​GGA​CAT​CA-3′, Cyclin A fw: 5′-GGA​AAT​TGC​AGC​TTG​TCG​GG-3′, Cyclin A rev: 5′-GCT​GTC​GCT​TTG​TGT​ACG​TG-3′, Cyclin B fw: 5′-CAG​ACG​ATG​GTG​GTG​ATC​CAA-3′, Cyclin B rev: 5′-TCC​AGT​GAC​TTC​ACG​ACC​CA-3′, and Ppia fw: 5′-CTG​AGC​ACT​GGG​GAG​AAA​GG-3′, Ppia rev: 5′-CAC​CCT​GGC​ACA​TGA​ATC​CT-3’. Gene expressions were normalized to Ppia expression.

### Western Blot

Total protein was isolated from cultured cells using RIPA buffer with added protease (Roche, 06538282001) and phosphatase inhibitors (Fisher Scientific, 502306746). Protein concentrations were quantified using a BCA assay (Pierce, PI23225) and equal amounts of protein were loaded for SDS-PAGE. GAPDH (Fitzgerald Industries International, 10R-G109a, 1:5000 dilution), p21 (Abcam, ab109199, and 1:500) and Erbb2 (Abcam, ab16901, 1:1000) primary antibodies and Anti-mouse IgG HRP-linked (Cell Signaling, 7076S) and Anti-rabbit IgG HRP linked (Cell Signaling, 7074S) secondary antibodies were used for blotting. Western blots were imaged after enhanced chemiluminescence using a Chemidoc station (Biorad).

### Statistical Analysis

All statistical comparisons were done using two tailed t-test. A *p* value below 0.05 was considered significant. Star annotations of ∗, ∗∗, ∗∗∗ are used to indicate *p* values below 0.05, 0.01 or 0.001, respectively.

## Results

### Rat Neonatal Cardiomyocytes

**FIGURE 1:**
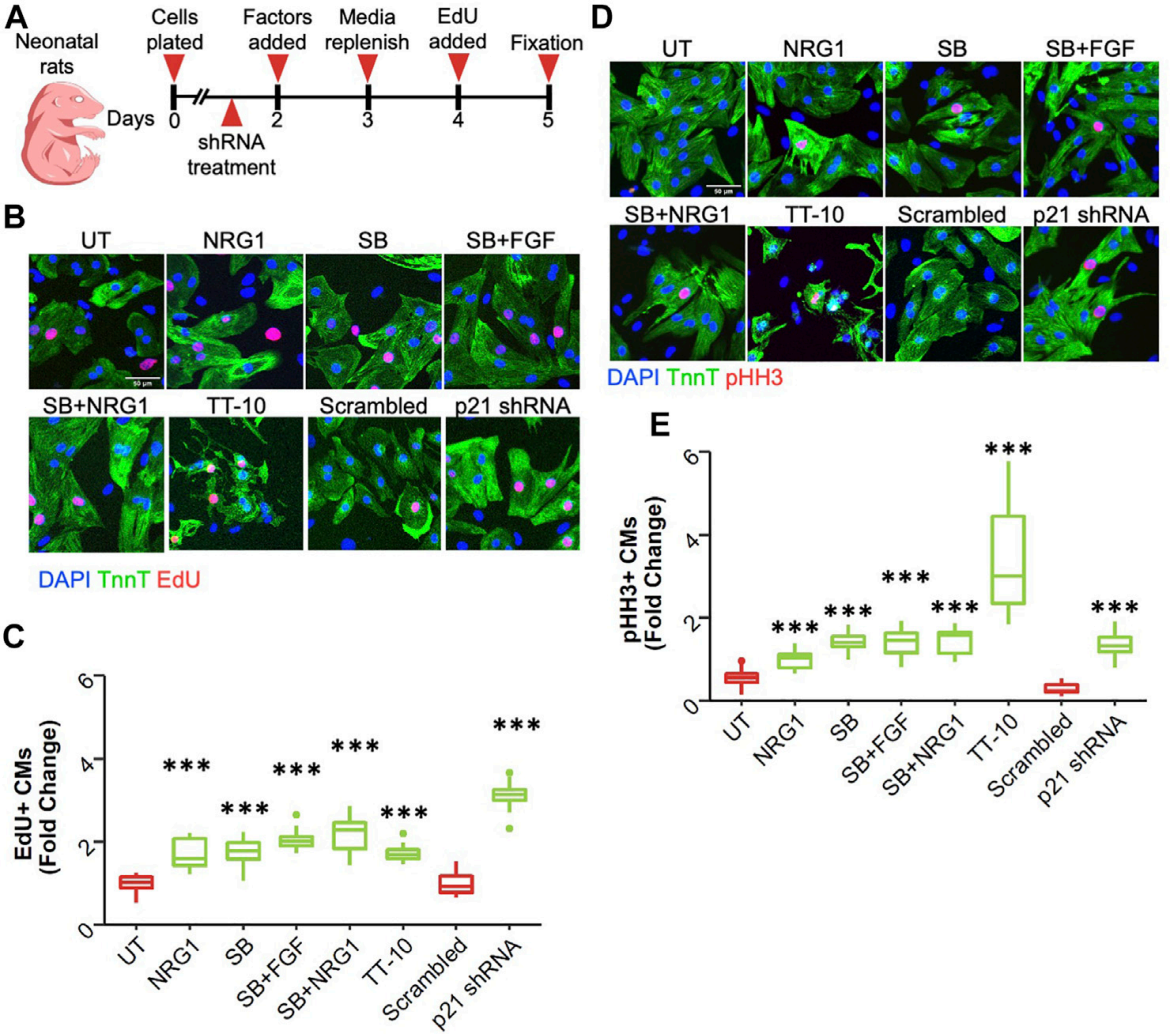
Stimulation of cardiomyocyte DNA synthesis. **(A)** Graphical overview of experimental outline. shRNAs are p21shRNA or scrambled shRNA adenoviruses. Other conditions are growth factors that were added on Day 2. **(B)** Representative images of EdU stained (red) cardiomyocytes (green) in response to various treatments to induce cardiomyocyte proliferation (green: Troponin T, red: EdU, blue: DAPI, scale bar equals 50 µm) **(C)** Percentage of EdU positive cardiomyocyte nuclei relative to total cardiomyocyte nuclei. ***p < 0.001 vs respective controls, n = 12 for each group except p21 shRNA (*n* = 11) and TT-10 (*n* = 10). **(D)** Representative images of pHH3 staining (green: Troponin T, red: pHH3, blue: DAPI, scale bar equals 50 µm) **(E)** Percentage of pHH3-positive cardiomyocyte nuclei relative to total cardiomyocyte nuclei. ***p < 0.001 vs respective control, *n* = 12 for each group except p21 shRNA (*n* = 11) and TT-10 (*n* = 10).

To study the extent of their proliferative capacities, we picked 6 conditions that have previously been shown to enhance cardiomyocyte proliferation. These stimulators include Neuregulin 1 (NRG1) (100 ng/ml), the MAPK p38 inhibitor SB203580 (SB) (10 µM), a combination of Fibroblast Growth Factor 1 (FGF) (50 ng/ml) with SB203580, Neuregulin 1 combined with SB203580, TT-10 (5 µM) and shRNA mediated knockdown of Cdkn1a (p21), which was validated by qPCR and western blotting (Supplementary Figures S1A,B) (Engel et al., 2006; Hara et al., 2018; Bersell et al., 2009). We isolated cardiomyocytes from neonatal rats and treated them with previously mentioned stimulators to induce cardiomyocyte proliferation as depicted in the schematic in Figure 1A. The thymidine analog EdU was added during the last 24-h of the culture to measure DNA synthesis. Representative images show EdU incorporation, indicative of DNA synthesis, in cardiomyocytes treated with the proliferative stimuli (Figure 1B). Using an adapted version of a previously published automated image analysis algorithm, we calculated the percentage of EdU positive cardiomyocyte nuclei by co-staining for troponin T to mark cardiomyocytes (Bass et al., 2012). All treatments resulted in significant increases in cardiomyocyte DNA synthesis compared to control conditions, with p21 shRNA showing the largest effect size (Figure 1C). To measure progression of DNA synthesis to mitosis, we measured phosphorylation of Histone H3, a well-known marker of chromosome condensation. Representative images of staining are shown in Figure 1D. All treatments significantly stimulated phosphorylation of Histone H3, indicative of progression of DNA synthesis to mitosis, with TT-10 showing the largest effect size (Figure 1E).

**FIGURE 2:**
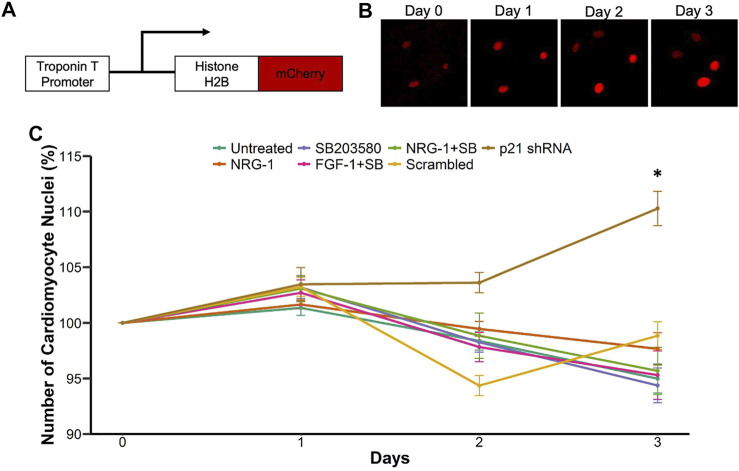
Live-cell imaging platform to quantify cardiomyocyte proliferation. **(A)** Schematic representation of Troponin T promoter driven Histone H2B-mCherry expressing adenovirus **(B)** Example of cardiomyocyte nuclear division **(C)** Live cell imaging mediated measurement of cardiomyocyte mitosis. Cell growth percentage is measured as number of cardiomyocyte nuclei at days 1–3 relative to day 0. ***p < 0.001 vs respective control, *n* = 12 for each group.

Next, we performed live-cell imaging to accurately quantify cardiomyocyte mitosis. To that end, we constructed an adenovirus expressing a cDNA for histone H2B fused with mCherry under control of the rat cardiomyocyte troponin T promoter (Figure 2A). Although this promoter fragment has previously been used to obtain cardiomyocyte-specific expression, we first validated that we indeed obtained exclusive expression of mCherry tagged histone H2B within cardiomyocytes (Supplementary Figure S2A). We determined an MOI of 25 as the least amount of virus to achieve efficient labeling. This gave rise to labeling of 94% of cardiomyocytes, while higher MOIs did not further increase this percentage (Supplementary Figure S2B). Next, we validated that we could use this technique to monitor and quantify the number of cardiomyocyte nuclei over the course of multiple days. We readily detected cardiomyocyte nuclei in live cardiomyocyte cultures with minimal exposure settings and could detect the new formation of cardiomyocyte nuclei over time (Figure 2B). Having established and validated a cardiomyocyte specific method to quantify cardiomyocyte mitosis over time, we next tested the various proliferative stimuli using this live-cell imaging platform. We measured the number of cardiomyocyte nuclei over the course of 3 days and plotted the percent change in cardiomyocyte nuclei number at each day. Interestingly, although all treatments showed enhanced EdU incorporation and pHH3 expression (Figure 2C), we did not detect an increase in cardiomyocyte nuclei for most conditions including NRG1 or SB. In contrast, p21 knockdown increased cardiomyocyte nuclei numbers over time compared to scrambled control (Figure 2C). To understand why p21 shRNA would give rise to more cardiomyocyte nuclei, but other conditions failed despite stimulation of EdU incorporation and phospho Histone H3 positive staining, we assessed the transcriptional level of mitotic cyclins A and B (Supplementary Figures S2C,D). Interestingly, we observed increased cyclin A and B mRNA expression only for p21 shRNA treated samples, while treatments involving NRG1, and SB failed to show differences compared with controls. This was also consistent with previous studies related to cardiomyocyte proliferation and cyclin A activity (Chaudhry et al., 2004; Shapiro et al., 2014). To further explore a potential explanation for the lack of cardiomyocyte mitosis for most conditions, given the significant increase in EdU and pHH3 staining, we hypothesized that some treatments might confer protection from cell death in these primary cell cultures. To test for that possibility, we performed live/dead assays using calcein and ethidium-homodimer (EthD-1) and quantified the cardiomyocyte nuclei positive for EthD-1 with low/no calcein signal (Supplementary Figure S2E). Interestingly, all SB203580 related treatments conferred protection against cell death, in the absence of added stimuli for cell death (Supplementary Figure S2F). Notably, in this experiment cells were fixed well before we added EdU in the end-point experiments to analyze cell death susceptibility at earlier points in the culture.

### Mouse Embryonic Cardiomyocytes

**FIGURE 3:**
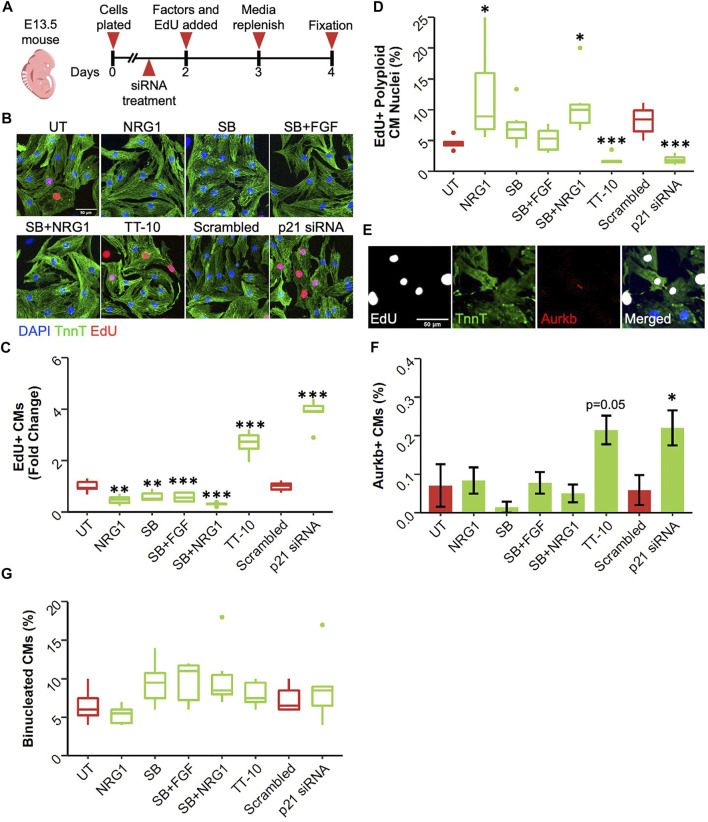
Effect of proliferative stimuli on mouse E13.5 cardiomyocytes. **(A)** Experimental outline. E13.5 mouse cardiomyocytes were isolated and plated on Day 0, 36 h later siRNAs and 48 h later proliferative stimuli were added. Edu was added along with proliferative stimuli and was present for the last 48 h of culture. **(B)** Representative images of E13.5 murine cardiomyocytes stained for TnnT (green), EdU (Red) and DAPI (blue) (scalebar = 50 µm) **(C)** Quantification of Fold Change in relative EdU + CM nuclei (*n* = 6 for each group, **p < 0.01, ***p < 0.001) **(D)** Quantification of EdU + polyploid (4n+) nuclei based on DAPI histograms (*n* = 6, *p < 0.05, ***p < 0.001) **(E)** Representative image of Aurkb + cardiomyocyte in the process of undergoing cytokinesis **(F)** Abundance of Aurkb + CMs relative to total number of CMs after treatments (*n* = 6 for each group, *p < 0.05) **(G)** Percentage of binucleated CMs at the end of culturing period (*n* = 6 for each group).

Although neonatal rat ventricular cardiomyocytes are a valuable source to study cardiomyocyte proliferation *in vitro*, mouse cardiomyocytes are also widely used due to the accessibility of various genetic mouse models. We used cardiomyocytes isolated at different developmental stages since the rate of cardiomyocyte proliferation varies greatly during early and late embryonic development (Uygur and Lee, 2016). First, we isolated cardiomyocytes from embryos at embryonic day 13.5 (E13.5), where cardiomyocytes are highly proliferative, and treated isolated cardiomyocytes with stimuli (Figure 3A). Due to the low baseline proliferation rate of mouse cardiomyocytes compared with rat cardiomyocytes, we administered EdU for the last 48 h of culture along with the proliferative stimuli to capture all DNA synthesis events happened during that period. We used immunocytochemistry to stain for troponin T and incorporated EdU and analyzed the percentage of EdU positive cardiomyocytes (Bass et al., 2012) (Figures 3B,C). Similar to neonatal rat ventricular cardiomyocytes, both TT-10 and p21 siRNA treatments stimulated DNA synthesis in early embryonic cardiomyocytes compared with their respective controls. Surprisingly, we observed a slight but significant reduction in cardiomyocyte DNA synthesis after treatments involving NRG1, SB and other combinations of SB (Figure 3C). To determine if the DNA synthesis stimulatory effects of TT-10 and p21 inhibition would lead to cardiomyocyte proliferation and new cell formation, we used a series of experiments. First, we used a previously published imaging algorithm to measure DAPI intensity to determine the amount of DNA in each cardiomyocyte nucleus (Yücel et al., 2020). As expected, we were able to separate diploid (2n) and polyploid (4n) cardiomyocyte nuclei successfully (Supplementary Figure S3). By selecting only EdU + CM nuclei, we specifically analyzed the fate of cardiomyocytes that were actively cycling during the last 48 h of culture. It is important to note that 4n cardiomyocyte nuclei fraction can represent both cells that are stuck or currently in G2 phase. However, since EdU was present for 48h in culture and considering the fact that G2/M phase only takes ∼4 h to complete, we assume the majority of the identified 4n cardiomyocyte nuclei represent the fraction that failed to enter mitosis after DNA replication. Analysis of the ploidy level per CM nucleus indicated that both TT-10 and p21 siRNA treatments decreased the fraction of polyploid CM nuclei, indicating that these treatments not only initiate cell cycle and but also cause completion of mitosis (Figure 3D). On the other hand, treatments such as NRG1 and the combination of NRG1 with SB resulted in increased frequency of EdU + polyploid CM nuclei. Cardiomyocytes can exit cell cycle after mitosis, prior to completion of cytokinesis, especially in mouse hearts (Patterson et al., 2017). To assess if the proliferative effects of TT-10 and p21 siRNA treatments would lead to actual cardiomyocyte cell division, we stained cardiomyocytes for the widely used telophase marker Aurora Kinase b (Aurkb), which localizes to the cleavage furrow during cytokinesis. We imaged and quantified Aurkb localization between 2 CMs to represent cytokinesis events (Figure 3E). Both TT-10 and p21 siRNA treatments increased the percentage of Aurkb positive cardiomyocytes (Aurkb + CMs), although the *p* value for TT-10 treatment was not quite significant (Figure 3F). None of the other factors increased the number of cardiomyocyte cell divisions. Furthermore, we failed to observe differences in the percentage of binucleated cardiomyocytes for any of the treatments, suggesting that early embryonic cardiomyocytes in culture primarily exit cell cycle in the G2 phase, prior to mitosis (Figure 3G).

**FIGURE 4:**
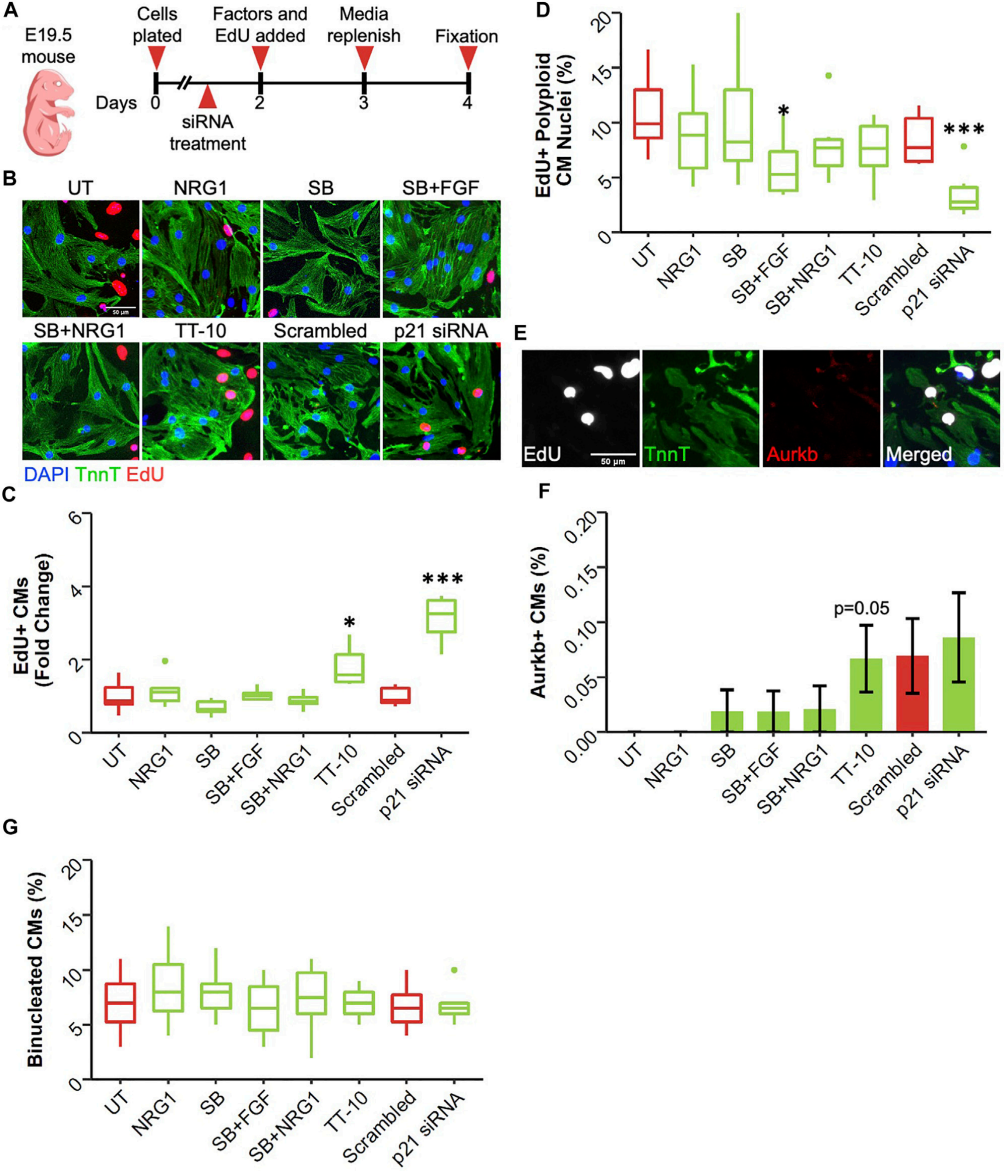
Effect of proliferative stimuli on mouse E19.5 cardiomyocytes. **(A)** Experimental outline. E19.5 mouse cardiomyocytes were isolated and plated on Day 0, 36 h later siRNAs and 48 h later proliferative stimuli were added. Edu was added along with proliferative stimuli and was present for the last 48 h of culture. **(B)** Representative images of embryonic cardiomyocytes stained for TnnT (green), EdU (Red) and DAPI (blue) (scalebar = 50 µm) **(C)** Quantification of Fold Change in relative EdU + CM nuclei (n = 6 for each group, *p < 0.05, ***p < 0.001) **(D)** Quantification of EdU + polyploid (4n+) nuclei based on DAPI histograms (*n* = 6, *p < 0.05, ***p < 0.001) **(E)** Representative image of Aurkb + cardiomyocyte in the process of undergoing cytokinesis **(F)** Abundance of Aurkb + CMs relative to total number of CMs after treatments (n = 6 for each group) **(G)** Percentage of binucleated CMs at the end of culturing period (n = 6 for each group).

Even during embryonic development, the rate of cardiomyocyte proliferation varies greatly between different timepoints. Moreover, the primary source of newly generated cardiomyocytes changes from cardiac progenitors in early development, to a small subset of cardiomyocytes in late development. To assess if the treatments have different effects at later stages of cardiac development, we isolated cardiomyocytes embryonic day 19.5, 1 day before birth (E19.5) (Figure 4A). Similar to E13.5 cardiomyocytes, only TT-10 and p21 siRNA treatments stimulated DNA synthesis measured by EdU positivity (Figures 4B,C). However, TT-10 treatment failed to induce DNA synthesis under serum free conditions, suggesting proliferative effects of TT-10 partially depends on the presence of serum (Supplementary Figure S4A **)**. Furthermore, the anti-proliferative effects of NRG1, SB and other combinations of SB that were observed in E13.5 were not present in E19.5 cardiomyocytes cultured with or without serum, suggesting these molecules might have a distinct effect on immature cardiomyocytes (Figure 4C; Supplementary Figure S4A). Next, we measured the rate of endoreplication in proliferating cardiomyocytes using DAPI intensity (Supplementary Figure S4B). Similar to E13.5 cardiomyocytes both TT-10 and p21 siRNA treatments decreased the rate of polyploid CMs in both serum free and with serum conditions, although the effect of TT-10 in presence of serum was not significant (Figure 4D; Supplementary Figure S4C **)**. Notably, the combination of SB with FGF also decreased the number of polyploid CMs, suggesting that even though p38 inhibition does not stimulate cell cycle entry, it might induce the completion of mitosis (Figure 4D; Supplementary Figure S4C). Next, we quantified cardiomyocyte cytokinesis after treatments using Aurora Kinase B (Figure 4E). Although TT-10 and p21 siRNA treatments induced cell cycle entry and M phase progression, only TT-10 to induce new cardiomyocyte formation at this timepoint (Figure 4F). Similar to the E13.5 timepoint, none of the conditions induced cardiomyocyte binucleation (Figure 4G).

### Mouse Postnatal Day 1 Cardiomyocytes

**FIGURE 5:**
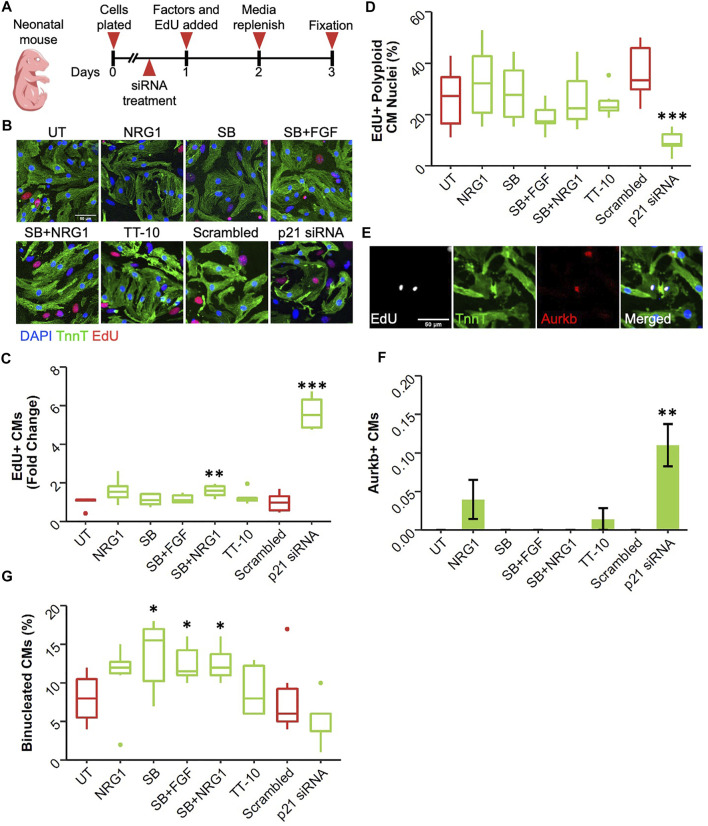
Effect of proliferative stimuli on neonatal mouse cardiomyocytes. **(A)** Experimental outline. Neonatal mouse cardiomyocytes were isolated and plated on Day 0, 36 h later siRNAs and 48 h later proliferative stimuli were added. Edu was added along with proliferative stimuli and was present for the last 48h of culture. **(B)** Representative images of neonatal mouse cardiomyocytes stained for TnnT (green), EdU (Red) and DAPI (blue) (scalebar = 50 µm) **(C)** Quantification of Fold Change in relative EdU + CM nuclei (*n* = 6 for each group, *p < 0.05, ***p < 0.001) **(D)** Quantification of EdU + polyploid (4n+) nuclei based on DAPI histograms (*n* = 6, *p < 0.05, ***p < 0.001) **(E)** Representative image of Aurkb + cardiomyocyte in the process of undergoing cytokinesis **(F)** Abundance of Aurkb + CMs relative to total number of CMs after treatments (*n* = 6 for each group) **(G)** Percentage of binucleated CMs at the end of culturing period (*n* = 6 for each group).

Cardiomyocyte proliferation declines dramatically soon after birth in mammalian hearts (Uygur and Lee, 2016). Therefore, it can be argued that postnatal day1 serves as a valuable timepoint as cardiomyocytes begin to lose their proliferative capacities at this stage. Using the same timeline as E13.5 and E19.5 cardiomyocyte cultures, we assessed the proliferative effect of the factors on neonatal cardiomyocytes (Figure 5A). Interestingly, at this timepoint, only p21 siRNA was effective in enhancing DNA replication in cardiomyocytes in both serum and no serum conditions (Figures 5B,C). Notably the combination of SB and NRG1 was able induce a slight but significant increase in the number of EdU + CMs in presence of serum (Figure 5C). p21 siRNA decreased the frequency of polyploid CM nuclei determined from DAPI histograms in presence of serum (Figure 5D; Supplementary Figure S5B). In contrast, we failed to observe this effect in serum free cultures, suggesting mitotic activity after p21 inhibition depends on serum (Supplementary Figure S5C). Next, we quantified the fraction of cardiomyocytes undergoing cytokinesis (Figure 5E). Only p21 siRNA treatment increased the frequency of dividing cardiomyocytes (Figure 5F). Surprisingly, SB and its combinations significantly increased the frequency of binucleated cardiomyocytes despite having little to no effect on cell cycle entry (Figure 5G).

### Mouse Adult Cardiomyocytes

**FIGURE 6:**
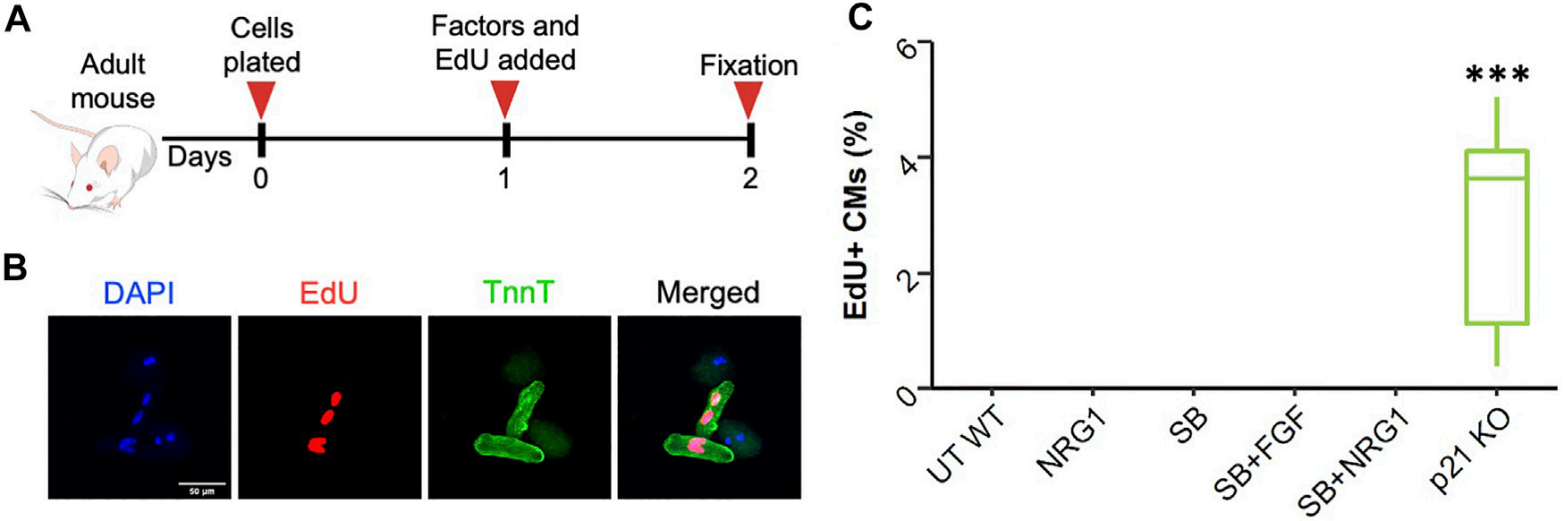
Effect of proliferative stimuli on adult mouse cardiomyocytes. **(A)** Schematic overview of the experimental outline. **(B)** Representative images of wild type and p21KO adult cardiomyocytes. Troponi T (green), EdU (red), DAPI (blue), scale bar equals 100 µm **(C)** Quantification of EdU + adult mouse cardiomyocytes 24 h after treatments. ***p < 0.001. (*n* = 9 for each group).

In later stages of postnatal development, cardiomyocytes almost completely lose their ability to enter cell cycle. To measure the cell cycle stimulatory effects at this developmental stage, we isolated adult cardiomyocytes from 3-month-old C57Bl/6j and p21 knockout (p21 KO) mice (Figure 6A). After isolation and 24 h of treatment, we did not detect any changes in cardiomyocyte DNA synthesis for any of the proliferative stimuli except for p21 deletion (Figures 6B,C). Adult cardiomyocytes isolated from p21KO mice showed enhanced EdU incorporation although we failed to observe any cytokinesis events, suggesting p21 deletion alone is insufficient to generate new cardiomyocyte formation (data not shown). Moreover, these results indicate that SB, FGF and NRG1 treatments are unable to induce DNA synthesis in freshly isolated adult cardiomyocytes.

### Human iPSC-Cardiomyocyte DNA Synthesis

**FIGURE 7:**
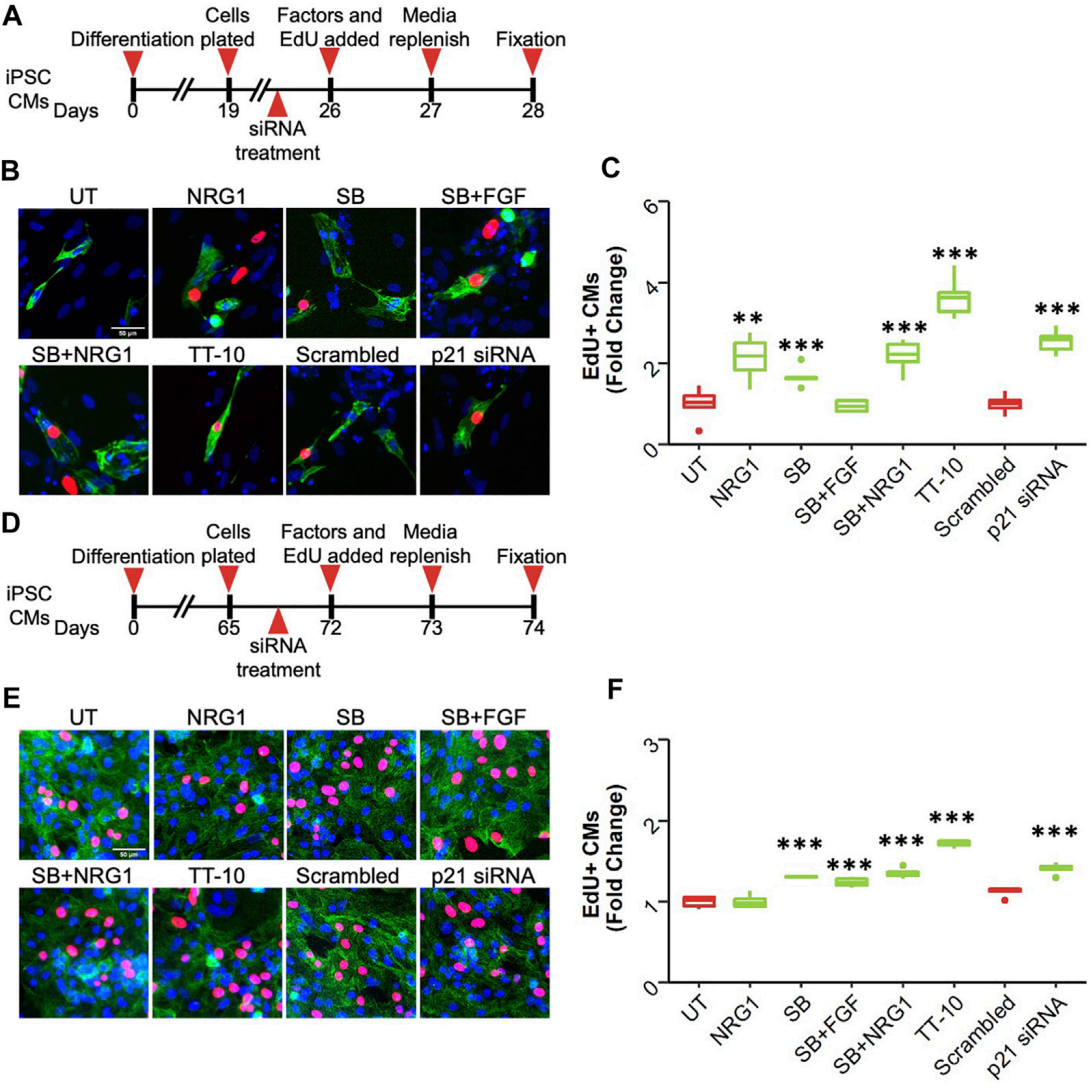
Effect of proliferative stimuli on human induced pluripotent stem cell derived cardiomyocytes (hiPSC-CMs) **(A)** Schematic overview of the experiment involving immature hiPSC-CMs. **(B)** Representative images of EdU + immature hiPSC-CMs after each treatment. Troponin T (green), EdU (red), DAPI (blue), scale bar equals 50 µm (*n* = 6, **p < 0.01, ***p < 0.001) **(C)** Quantification of EdU + immature hiPSC-CMs 48 h after treatment. *p < 0.05 ***p < 0.001 **(D)** Schematic overview of the experiment involving mature hiPSC-CMs. **(E)** Representative images of EdU + mature hiPSC-CMs after each treatment. Troponin T (green), EdU (red), DAPI (blue), scale bar equals 50 µm **(F)** Quantification of EdU + mature hiPSC-CMs 48 h after treatment. *p < 0.05 ***p < 0.001 (*n* = 6 for each group).

Finally, we measured the proliferative effects of these treatments on human induced pluripotent stem cell-derived cardiomyocytes (hiPSC-CM). We used three different cell lines that varied in terms of maturation and confluency. As the first source, we used immature hiPSC-CMs, where differentiation was initiated 26 days prior to the assessment of the proliferative effects of stimuli. We added the proliferative stimuli or p21 siRNA and incubated them for 48 h in the presence of EdU (Figure 7A). Interestingly, most proliferative factors, other than the combination of SB and FGF1, were able induce cardiomyocyte cell cycle activation in these cells (Figures 7B,C). However, when we used more mature hiPSC-CMs that were at least 70 days post-differentiation, we were unable to visualize the proliferative effects of NRG1 while all the other factors enhanced hiPSC-CM proliferation (Figures 7D–F). Notably, other studies have also shown that NRG1 by itself is not enough to stimulate hiPSC-CM proliferation. We repeated our findings using a second mature hiPSC-CM line and observed only a slight increase in proliferation after NRG1 treatment while other treatments were highly effective (Supplementary Figures S7A,B **)**. These results suggested that NRG1-dependent signaling through Erbb2 might be affected in more mature hiPSC-CMs. To determine if Erbb2 is expressed in hiPSC-CMs we performed western blotting of hiPSC-CMs and compared expression to mouse hearts at E17.5 and adult (Supplementary Figures S7C,D). Surprisingly, the level of Erbb2 protein expression was similar to E17.5 lysates, suggesting the presence of Erbb2 protein might not be sufficient for NRG1 to stimulate cardiomyocyte proliferation in mature hiPSC-CMs.

## Discussion

Based on research in lower vertebrates and neonatal mice, the heart could be seen a highly regenerative organ (Porrello et al., 2011; Foglia and Poss, 2016). This was clearly demonstrated in neonatal mice that underwent apical resection, and more recently in adult mice with forced expression of Yes-Associated Protein 1 (Yap-1) or a combination of 4 cell cycle regulators. As long as cardiomyocytes retain the ability to proliferate, the heart can regenerate from a surgical injury. However, when apical resection is performed 7 days after birth, when cardiomyocytes have lost the ability to proliferate, and this results in the formation of a scar. Therefore, stimulation of cardiomyocyte proliferation is an important target to accomplish cardiac regeneration.

Here, we measured the proliferative effects of various previously published cardiomyocyte cell cycle enhancers on cardiomyocytes from different species and at different developmental stages to expand our knowledge. . Our results suggest that one important reason could be because the proliferative capacity of these factors was only investigated using a single cell source or a single timepoint in cell culture. It is important to note that many changes happen in both transcriptomic and proteomic profiles of cardiomyocytes throughout development. These developmental gene expression changes not only affect cardiomyocyte contractility but also their growth and proliferation potential. Furthermore, cardiomyocytes obtained from different species may also show differences in certain cellular functions. For example, the two commonly used sources of primary cardiomyocytes, rats and mice, have different mitochondrial function and reactive oxygen species (ROS) generation rates (Liu et al., 2019). Therefore, to identify the most potent proliferative stimulus it would be important to validate initial findings across different species.

For our study, we used Cdkn1a inhibition, TT-10, Neuregulin-1, MAPK p38 inhibitor SB203580, and its combinations with both Neuregulin-1 and FGF-1 as proliferative stimuli. We first tested these approaches using neonatal rat ventricular cardiomyocytes. All stimuli enhanced cardiomyocyte cell cycle activity to a similar degree measured by both S phase and mitotic entrance using EdU and pHH3, respectively. Of note, the observed levels of phosphorylated histone H3 were much lower compared with EdU incorporation rates. This is likely a reflection of the cumulative nature of EdU incorporation compared with the transitory phosphorylation of Histone H3 at the onset of mitosis, combined with the longer duration of S-phase, ∼8 hrs, vs. G2/M, ∼4 h. Another potential explanation for this difference could be a cessation of cell cycle progression to mitosis despite completion of DNA synthesis (G2 arrest). Live cell imaging showed that the level of completion of mitosis varied considerably. In rat neonatal cardiomyocytes, only p21 inhibition was able to stimulate cardiomyocyte mitosis, which was further corroborated by enhanced Cyclin A and B expression only when p21 was knocked down, although we did not test TT-10 using live-cell imaging.

To further expand our knowledge on these proliferative stimuli, we included mouse cardiomyocytes from different embryonic and postnatal developmental timepoints. Surprisingly, treatments involving NRG1, and SB had little to no effect on mouse cardiomyocyte proliferation *in vitro* in any of the timepoints. Contrary to our expectations, these treatments actually reduced cardiomyocyte proliferation at an early developmental timepoint (E13.5). New cardiomyocyte formation is likely derived from a mix of cardiac progenitor cell and cardiomyocyte proliferation in early embryonic timepoints (Sereti et al., 2018). It is possible that these factors reduce the proliferation or differentiation rates of the progenitors, consequently reducing new cardiomyocyte formation. However, when we assessed their ability to stimulate proliferation at later stages including adulthood, we also failed to observe proliferative effects. It is possible that the signaling pathways through which these factors exert their proliferative effects are more abundant in rat cardiomyocytes than in mouse, thereby stimulating cell cycle entry only in rat cardiomyocytes. However, when we assessed Erbb2 expression in mouse hearts at an embryonic timepoint, we could readily detect its expression, suggesting that NRG1 signaling should be possible.

Among the tested stimuli, 2 factors consistently induced mouse cardiomyocyte proliferation. These factors were TT-10, a Yap1-TEAD activator and p21 inhibition, although TT-10 was less effective at neonatal stage and its proliferative effects were substantially lower in E19.5 compared with E13.5. Although Yap1 activation has been shown to be sufficient to drive cardiomyocyte proliferation in all developmental stages, it is unclear if TT-10 by itself is sufficient to drive proliferation in these timepoints (Heallen et al., 2011; Leach et al., 2017). It is possible that proteins such as Lats1 and Lats2 inhibit Yap1, and TT-10 might be insufficient to prevent this inhibition in certain stages of development. P21 inhibition induced cardiomyocyte DNA replication in all developmental stages we tested. In addition, p21 inhibition also increased completion of cardiomyocyte mitosis, confirmed by the reduction in the number of polyploid cardiomyocytes. However, we failed to observe cardiomyocyte cytokinesis at certain stages such as E19.5 and adulthood, suggesting that p21 inhibition by itself might be insufficient to achieve cardiac regeneration. To further explore the proliferative effect of the stimuli used, we included hiPSC-CMs. Although hiPSC-CMs are not considered mature under regular culture conditions, there is still a substantial difference between cardiomyocyte maturity in different stages after differentiation (Lewandowski et al., 2018; Guo and Pu, 2020). To have a better understanding of the proliferative effects of our stimuli, we included hiPSC-CMs from both day 28 (immature) and day 74 (mature) post-differentiation timepoints. Interestingly, although NRG1 by itself was able to induce cardiomyocyte proliferation in early immature hiPSC-CMs, it was less effective in two different mature hiPSC-CM lines, suggesting that the effect of NRG1 on proliferation could diminish with advanced maturation. The lack of NRG1 in stimulating proliferation of hiPSC-CMs was also reported by another study (Ito et al., 2019). In contrast, all other tested factors stimulated hiPSC-CM proliferation.

For E19.5 and neonatal cardiomyocyte cultures, we have used both serum (2% FBS) and no serum conditions to examine the proliferative effects of our stimuli separately. Interestingly, TT-10 stimulated E19.5 cardiomyocyte proliferation only when it was administered in serum containing media. Moreover, although p21 inhibition mediates cardiomyocyte mitosis and reduces the number of polyploid neonatal cardiomyocytes in the presence of serum, this effect was diminished when serum was absent. Overall, these findings highlight the importance of evaluating the proliferative effects of molecules in absence and presence of additional growth factors. Arguably, it is also possible that serum free conditions reduce the survival of cardiomyocytes, therefore indirectly affecting their proliferative potential.

An important aid in assessing the true proliferative effect of a stimulus could come from live-cell imaging of cardiomyocyte mitosis and cytokinesis. We used live-cell imaging to assess cardiomyocyte mitosis and were able to show substantial differences in the ability of stimuli to induce completion of mitosis. The live-cell imaging approach we used has its limitations as it only visualizes and quantifies cardiomyocyte mitosis. It is well-established that cardiomyocytes can undergo endoreplication. To measure actual cytokinesis events, we used localization of Aurkb at the cleavage furrow in our study. However, due to the short duration of cardiomyocyte cytokinesis and the abundance of Aurkb + CMs being very low to non-existent in certain conditions, the results varied greatly between replicates. To mitigate the limitations of our live-cell imaging platform and Aurkb based quantification of cytokinesis, future studies could employ a membrane-targeted dye in combination with H2B-mCherry Adenovirus, thereby allowing visualization and quantification of both cardiomyocyte mitosis and cytokinesis. Finally, *in vivo* verification of any identified potential mediator of cardiomyocyte proliferation is important since the *in vivo* environment is different from cell culture and may impact the proliferative capacity.

In conclusion, it is important to validate potential regulators of cardiomyocyte proliferation with various assays and in potentially in different species as a first step towards developing new therapies that enhance cardiomyocyte proliferation as a regenerative strategy. Here, we tested various putative enhancers of cardiomyocyte proliferation and identified inhibition of cdkn1a/p21 and the Hippo pathway modulator TT-10 as the most promising ones.

## Data Availability

The raw data supporting the conclusion of this article will be made available by the authors, without undue reservation.
