# Supplementary material for: Stimulation of Cardiomyocyte Proliferation Is Dependent on Species and Level of Maturation
Source: Front Cell Dev Biol. 2022 May 19;10:806564. doi: 10.3389/fcell.2022.806564 (PMC9160302; doi:10.3389/fcell.2022.806564)
Supplement: Supplementary file 1 [file DataSheet1.PDF]

Supplementary Materials for

Stimulation of cardiomyocyte proliferation is dependent on species and level of maturation.

Doğacan Yücel[1,2], Bayardo I. Garay[1,3,4], Rita C.R. Perlingeiro[1,2,3,5], Jop H. van Berlo[1,2,5]

1. Lillehei Heart Institute, Cardiovascular Division, Department of Medicine, University of Minnesota, Minneapolis, MN
2. Department of Integrative Biology and Physiology, University of Minnesota, Minneapolis, MN
3. Department of Neuroscience, University of Minnesota, Minneapolis, MN
4. Medical Scientist Training Program, University of Minnesota Medical School, Minneapolis, MN
5. Stem Cell Institute, University of Minnesota, Minneapolis, MN

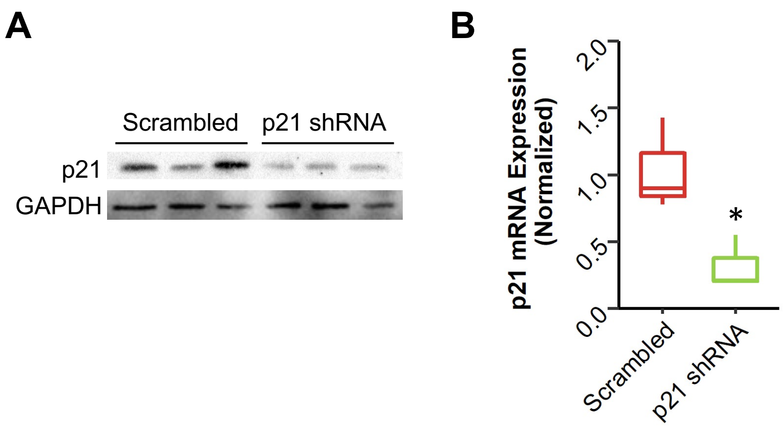

Supplementary Figure 1

(**A**) Representative Western blot for p21 and GAPDH showing knock-down of p21 protein in neonatal rat ventricular cardiomyocytes in response to lentiviral p21 shRNA transduction. (**B**) Quantitative PCR for Cdkn1a (p21), corrected to housekeeping gene PPIA from RNA extracted from cultured neonatal rat ventricular cardiomyocytes in response to scrambled shRNA or p21 shRNA expressing lentiviral transduction. *$p<0.05$ (n=3 for each group)

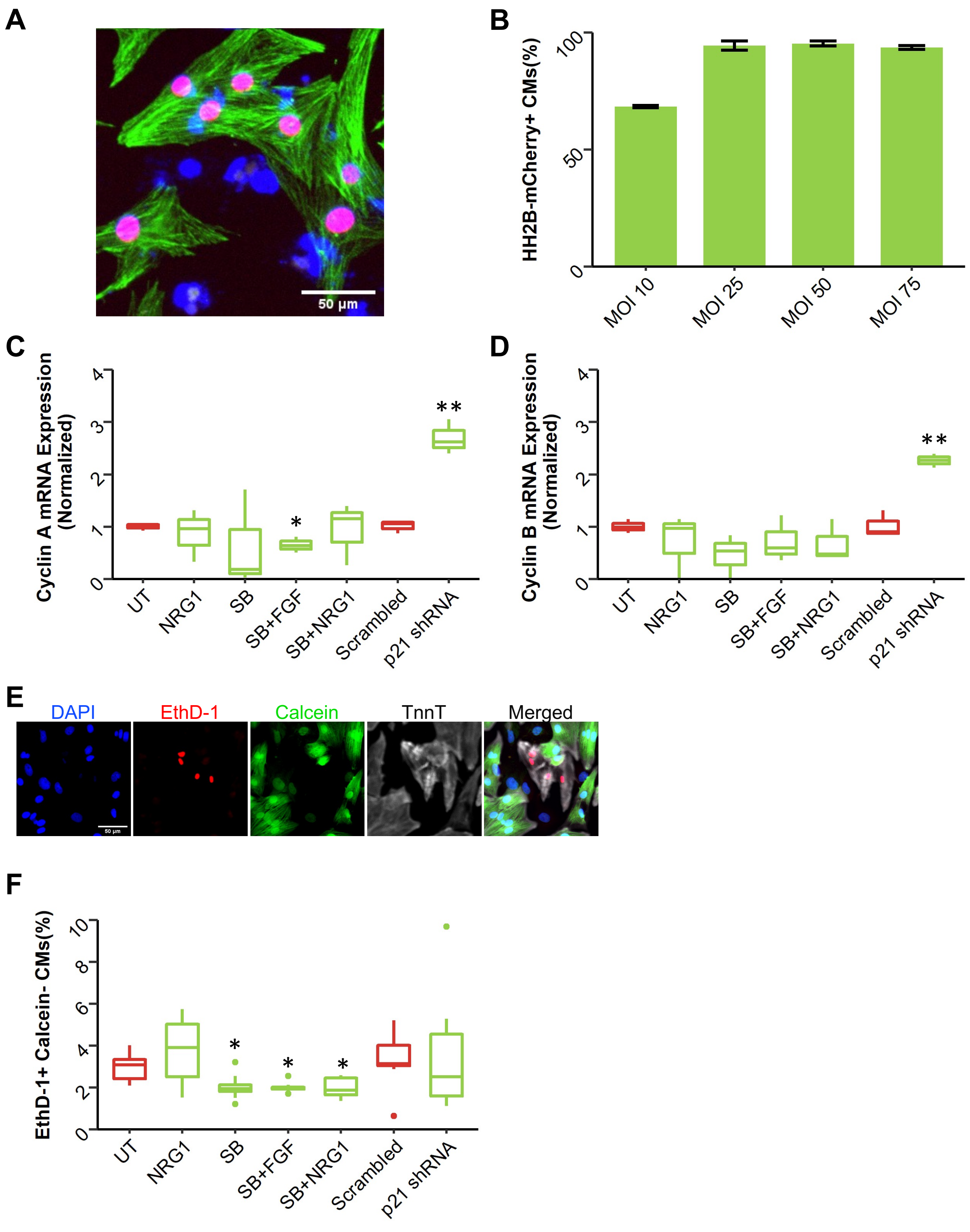

Supplementary Figure 2

(**A**) Representative image of rat neonatal ventricular cardiomyocytes infected with Histone H2B-mCherry virus expressing Histone H2B-mCherry (red) and stained with Troponin T (green) and DAPI (blue) (scale bar=50 μm) **(B)** Fraction of cardiomyocytes with detectable mCherry expression at indicated MOI. (n=4 for each sample) **(C-D)** Quantitative PCR for cell cycle regulators **(C)** Cyclin A and **(D)** Cyclin B on RNA extracted from cardiomyocytes treated as indicated. *$p<0.05$, **$p<0.01$ (n=3 for each group) **(E)** Representative image of staining for DAPI (blue), Ethidium homodimer (EthD-1, red), Troponin T (grey) and Calcein (green) to quantify live and dead/dying cardiomyocytes. Arrows point to cardiomyocyte nuclei that are positive for EthD-1, negative for Calcein AM (scale bar=50 μm) **(F)** Percentage of rat neonatal ventricular cardiomyocyte nuclei that are positive for EthD-1 and negative for calcein. Scale bar equals 50 μm *$p<0.05$ n=8 for each group

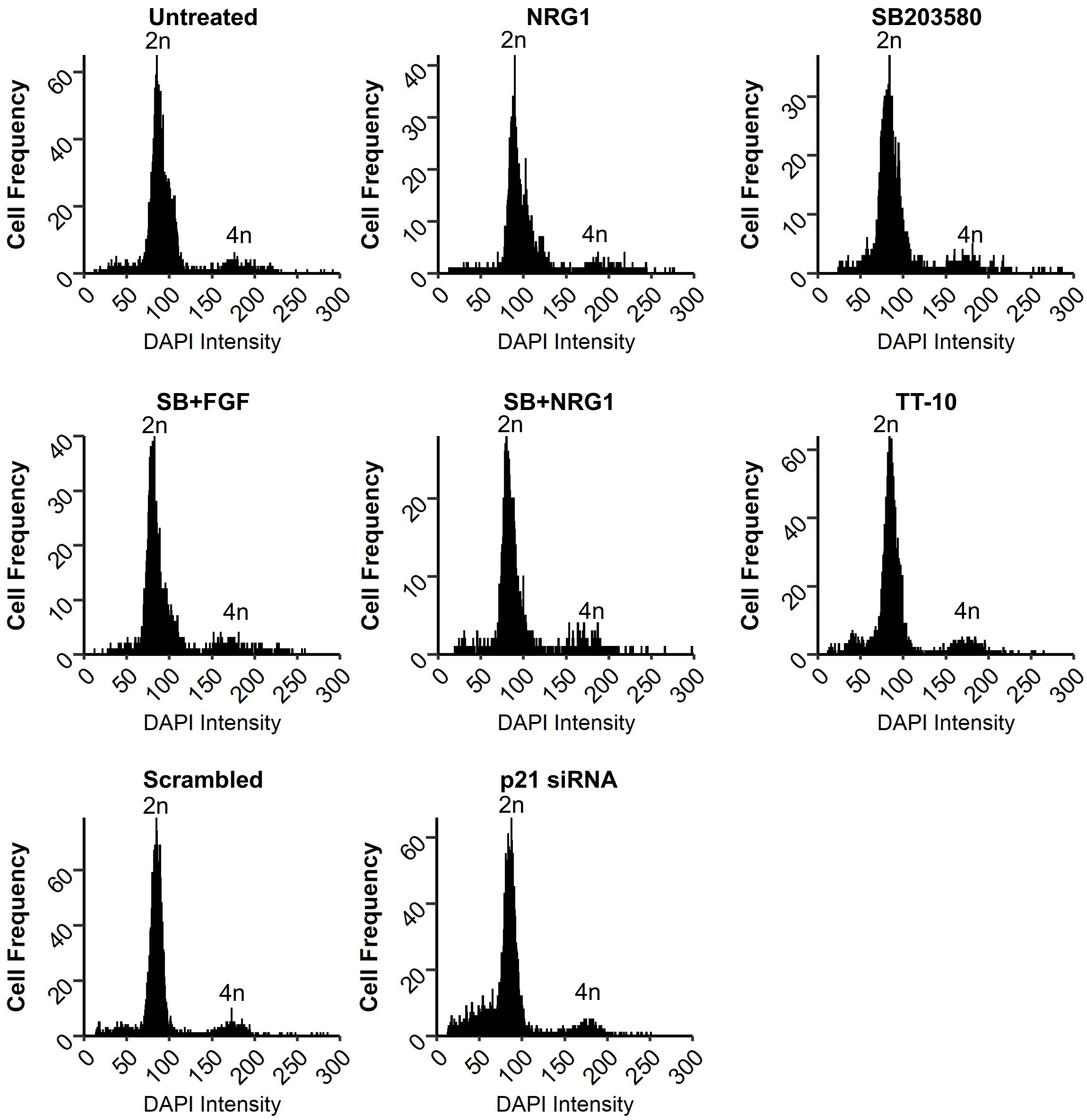

Supplementary Figure 3

Representative histograms for DAPI intensity of E13.5 mouse cardiomyocyte nuclei. 2n indicates diploid and 4n indicates polyploid CM nuclei

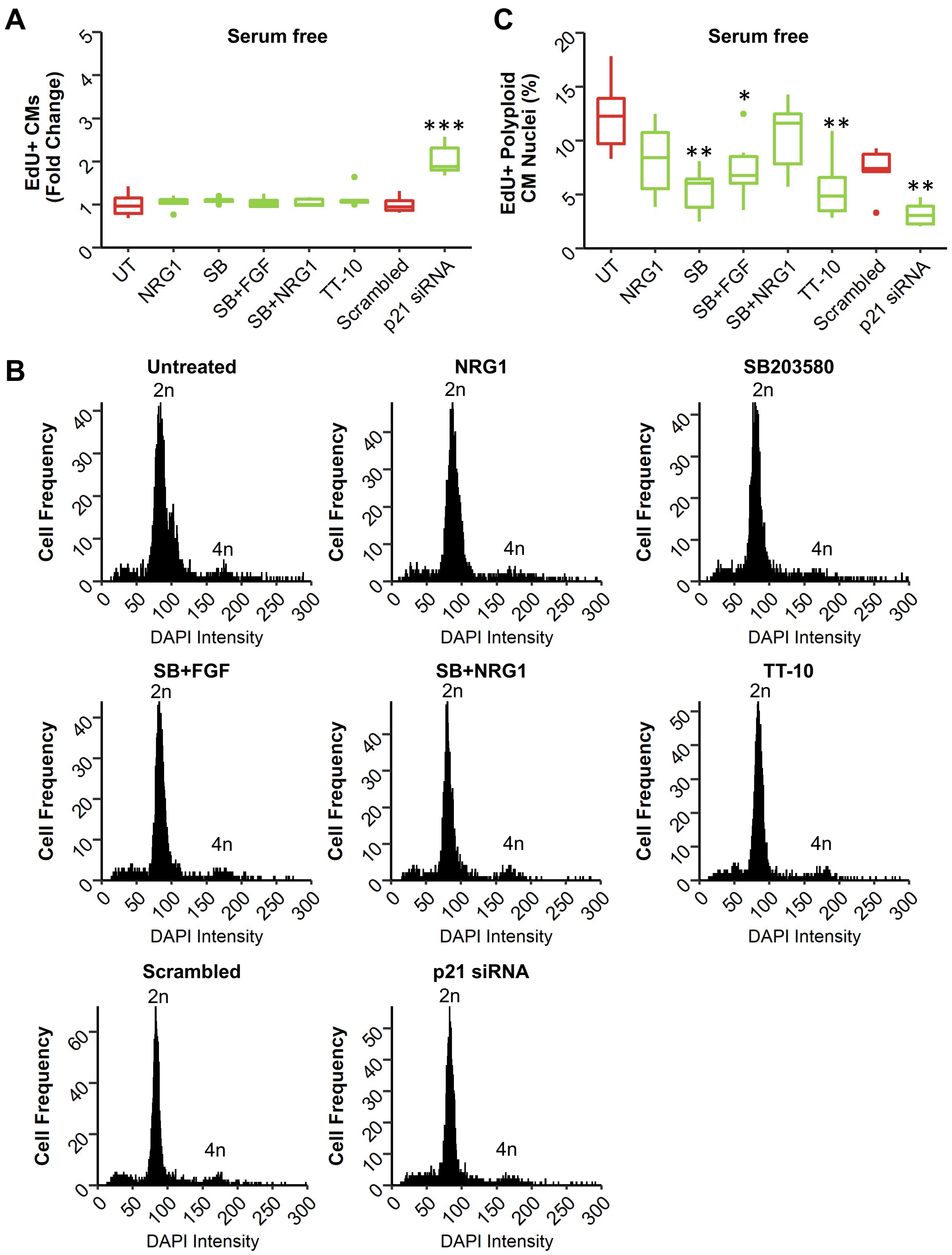

Supplementary Figure 4

(A) Relative number of EdU+ E19.5 mouse cardiomyocytes after each stimulus in serum free conditions (n=6, ***p<0.001) **(B)** Representative histograms for DAPI intensity of E19.5 cardiomyocyte nuclei. 2n indicates diploid and 4n indicates polyploid E19.5 mouse CM nuclei **(C)** The relative abundance of EdU+ polyploid E19.5 mouse CMs in serum free conditions (n=6, *p<0.05, **p<0.01)

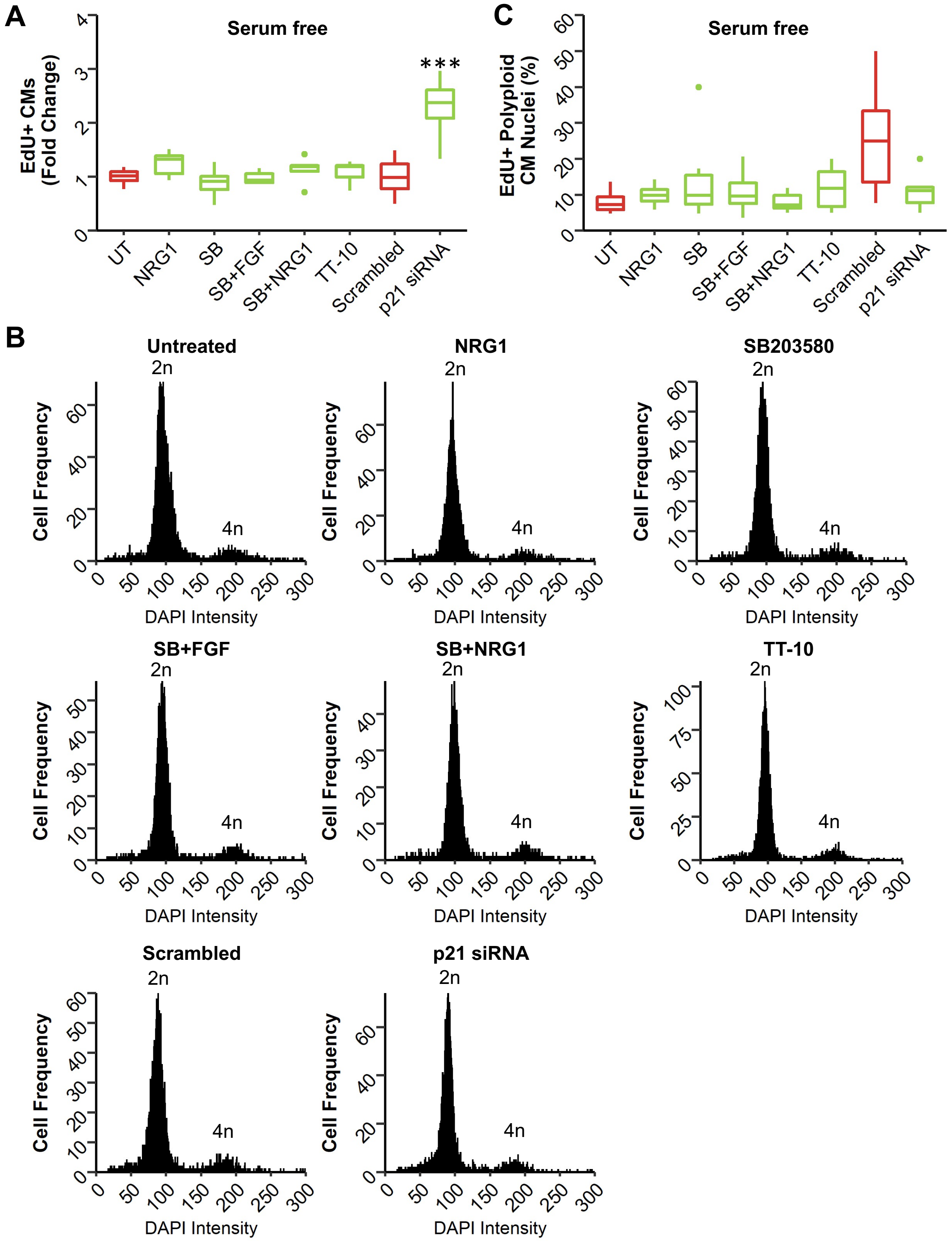

Supplementary Figure 5

(A) Relative number of EdU+ neonatal mouse cardiomyocytes after each stimulus in serum free conditions (n=6, ***p<0.001) **(B)** Representative histograms for DAPI intensity of neonatal mouse cardiomyocyte nuclei. 2n indicates diploid and 4n indicates polyploid CM nuclei **(C)** The relative abundance of EdU+ polyploid neonatal mouse CMs in serum free conditions (n=6)

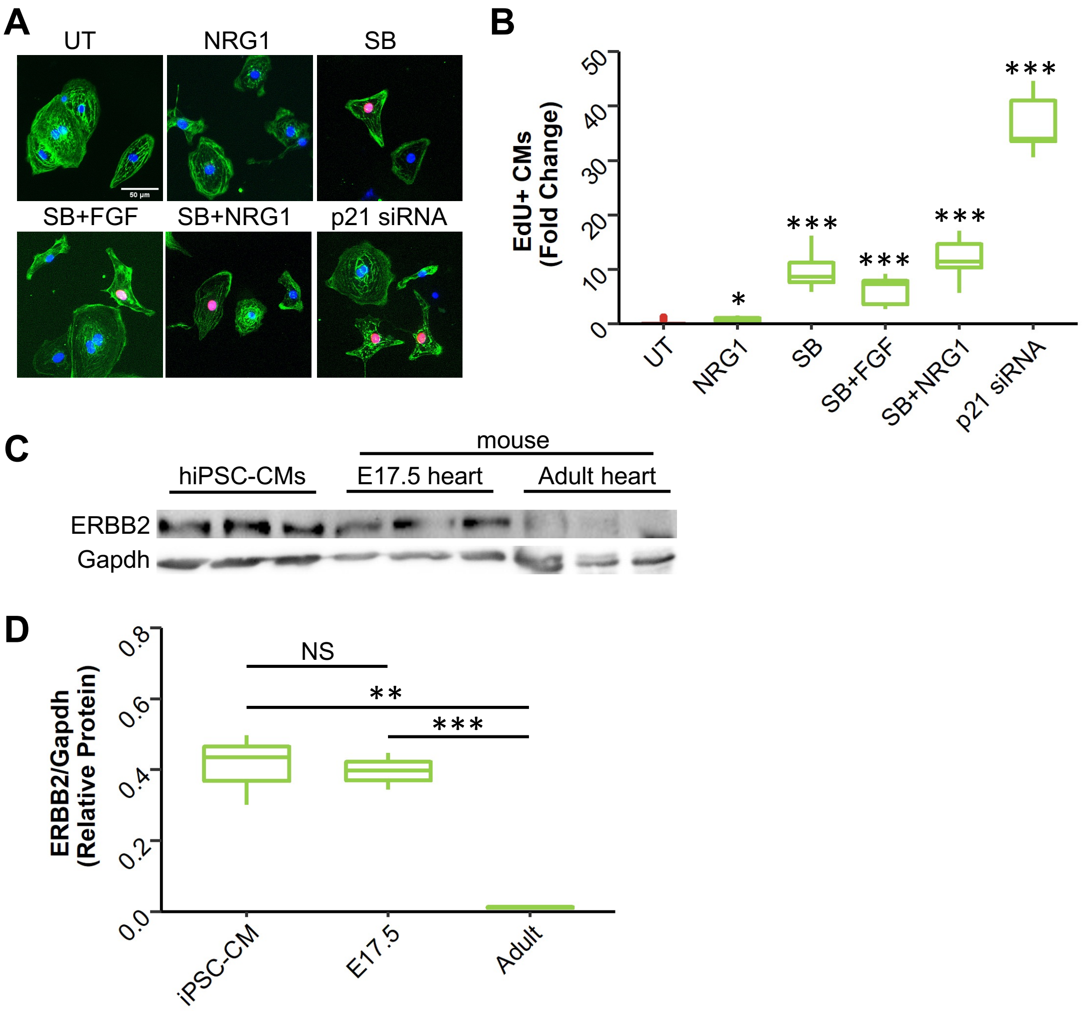

Supplementary Figure 6

(A) Representative images of EdU-stained mature hiPSC-CMs in low density culture. Troponin T (green), EdU (red), DAPI (blue), scale bar equals 50 μm **(B)** Relative abundance of EdU+ mature hiPSC-CMs after treatments (n=9 for each group, *p<0.05, ***p<0.001) **(C)** Western blot image of Erbb2 and Gapdh in mature hiPSC-CM, E17.5 and adult whole heart lysates. **(D)** Protein expression of Erbb2 in hiPSC-CM, E17.5 and adult whole heart lysates relative to Gapdh (n=3 for each group, **p<0.01, ***p<0.001)
